# Effects of Ocean Acidification on Resident and Active Microbial Communities of *Stylophora pistillata*

**DOI:** 10.3389/fmicb.2021.707674

**Published:** 2021-11-25

**Authors:** Marcelle Muniz Barreto, Maren Ziegler, Alexander Venn, Eric Tambutté, Didier Zoccola, Sylvie Tambutté, Denis Allemand, Chakkiath Paul Antony, Christian R. Voolstra, Manuel Aranda

**Affiliations:** ^1^Red Sea Research Center, Division of Biological and Environmental Science and Engineering (BESE), King Abdullah University of Science and Technology, Thuwal, Saudi Arabia; ^2^Department of Animal Ecology and Systematics, Justus Liebig University Giessen, Giessen, Germany; ^3^Center Scientifique de Monaco, Monaco, Monaco; ^4^Department of Biology, University of Konstanz, Konstanz, Germany

**Keywords:** climate change, coral microbiome, 16S rDNA, 16S rRNA, coral holobiont

## Abstract

Ocean warming and ocean acidification (OA) are direct consequences of climate change and affect coral reefs worldwide. While the effect of ocean warming manifests itself in increased frequency and severity of coral bleaching, the effects of ocean acidification on corals are less clear. In particular, long-term effects of OA on the bacterial communities associated with corals are largely unknown. In this study, we investigated the effects of ocean acidification on the resident and active microbiome of long-term aquaria-maintained *Stylophora pistillata* colonies by assessing 16S rRNA gene diversity on the DNA (resident community) and RNA level (active community). Coral colony fragments of *S. pistillata* were kept in aquaria for 2 years at four different *p*CO_2_ levels ranging from current pH conditions to increased acidification scenarios (i.e., pH 7.2, 7.4, 7.8, and 8). We identified 154 bacterial families encompassing 2,047 taxa (OTUs) in the resident and 89 bacterial families including 1,659 OTUs in the active communities. Resident communities were dominated by members of Alteromonadaceae, Flavobacteriaceae, and Colwelliaceae, while active communities were dominated by families Cyclobacteriacea and Amoebophilaceae. Besides the overall differences between resident and active community composition, significant differences were seen between the control (pH 8) and the two lower pH treatments (7.2 and 7.4) in the active community, but only between pH 8 and 7.2 in the resident community. Our analyses revealed profound differences between the resident and active microbial communities, and we found that OA exerted stronger effects on the active community. Further, our results suggest that rDNA- and rRNA-based sequencing should be considered complementary tools to investigate the effects of environmental change on microbial assemblage structure and activity.

## Introduction

Coral reefs are among the most biodiverse and productive ecosystems in the world. Despite covering less than 0.2% of the marine environment, they harbor 34% of the described marine biodiversity (Reaka-Kudla, 2001). Hundreds of millions of people depend on them for their livelihood, since they provide goods and services such as fisheries, tourism, coastal protection, pharmaceutical substances and aesthetic and cultural value (Moberg and Folke, 1999). The framework of reefs is built by scleractinian corals. They are referred to as holobionts, obligate symbiotic organisms that live in close association with microorganisms such as endosymbiotic photosynthetic algae, bacteria, archaea, viruses and fungi (Rohwer et al., 2002; Rosenberg et al., 2007). Many studies have looked into the symbiotic relationship with the dinoflagellate algae from the family Symbiodiniaceae (LaJeunesse et al., 2018), but less is known about the bacterial partners in this association. The coral microbiome has a range of functional roles, including nitrogen fixation (Lesser et al., 2004; Lema et al., 2012; Pogoreutz et al., 2017a,b), sulfur cycling (Raina et al., 2009), antibacterial activities (Ritchie, 2006) and overall holobiont health (Rosenberg et al., 2007; Robbins et al., 2019; Voolstra and Ziegler, 2020). As high throughput sequencing of microbial DNA becomes more accessible, studies based on analysis of phylogenetic markers such as the 16S rRNA gene become increasingly available.

Coral reefs are declining globally, mainly due to rising CO_2_ emissions (Hoegh-Guldberg, 2007; Hughes et al., 2017). Besides causing ocean warming, rising CO_2_ in the atmosphere is also linked to ocean acidification (OA), which is caused by elevated partial pressure of carbon dioxide (*p*CO_2_) in the seawater that alters carbonate ion chemistry and reduces seawater pH (Doney et al., 2009). Seawater surface pH has decreased 0.1 units since pre-industrial values and is predicted to decrease another 0.2–0.4 units by 2100 (Rhein, 2014). There is evidence that OA can negatively impact calcifying organisms, such as scleractinian corals, by decreasing their calcification rate and photosynthesis (Anthony et al., 2008; Doney et al., 2009). However, it is still not clear how OA might affect the coral microbiome, and consequently holobiont physiology and health. Some studies showed that OA can shift coral microbiome composition from a healthy-associated community to one usually seen in diseased and stressed corals (Thurber et al., 2009; Meron et al., 2011; Webster et al., 2013), while others show a stable microbiome (Meron et al., 2012; Webster et al., 2016) or species-specific responses (Morrow et al., 2015; Grottoli et al., 2018).

Most studies that characterized microbial community composition were based on 16S rDNA amplicon sequencing, an established method that can successfully describe microorganisms present in the community (Lane et al., 1985; Weisburg et al., 1991). However, one limitation of this method is that it includes any DNA, including DNA from dormant and dead microbes, making it impossible to distinguish the metabolically active from the non-active population (Pietramellara et al., 2009; Blagodatskaya and Kuzyakov, 2013; Carini et al., 2016). Alternatively, sequencing 16S rRNA serves as an effective proxy to estimate active microbial populations, since it is a component of ribosomes, which are predicted to increase in number with increasing metabolic activity (Poulsen et al., 1993). In addition, once outside the cell, RNA degrades faster than DNA, ensuring sampling of only the active community. Thus, the ratio of rRNA/rDNA of a specific taxon can be used as a measure of its potential activity (Jones and Lennon, 2010), although this approach is not without its caveats (Blazewicz et al., 2013). Many studies have pointed out a mismatch between the DNA/RNA based communities (DeAngelis and Firestone, 2012; Dlott et al., 2015; Steven et al., 2017). Hence, simultaneous sequencing and comparison of both components can unveil rare but highly active, as well as abundant and inactive taxa (Jones and Lennon, 2010; Campbell et al., 2011; Gaidos et al., 2011; Lanzén et al., 2011; Hunt et al., 2013).

In this study, we assessed the effect of long term increased *p*CO_2_ treatments on the microbial community of the Red Sea coral *Stylophora pistillata* at both the 16S rDNA and rRNA level to obtain an integrative view of the effects on the resident and active microbial partners.

## Materials and Methods

### Coral Husbandry

Nubbins of *Stylophora pistillata* have been kept in a long-term experimental aquarium setup since the early 2010’s at the Monaco Scientific Centre as described previously (Venn et al., 2013; Tambutté et al., 2015; Liew et al., 2018) and were sampled for this study in April 2014, after 2 years of OA treatment. Coral nubbins were exposed to four different *p*CO_2_ treatments: 2,447.48 ± 7.16, 2,461.77 ± 5.97, 2,474.16 ± 5.89 and 2,496.62 ± 6.99 μmol kg^–1^ total alkalinity. These treatments were equivalent to: pH 8.0 (control), 7.8, 7.4 and 7.2, respectively. Colonies were divided into four fragments and placed under the different treatments, with three fragments sampled from each treatment. Carbonate chemistry was manipulated by bubbling CO_2_ to decrease pH to the desired target values (*p*CO_2_ levels: 537.66 ± 4.88, 798.77 ± 14.36, 2109.3 ± 59.63, 3513.15 ± 67.20 μatm). Temperature and pH were monitored in the four treatment tanks with probes (Ponsel-Mesure, France) linked to a monitoring system (Enoleo, Monaco) that also controlled CO_2_ bubbling. Mediterranean water was supplied at an exchange rate of 70% h^–1^, adjusted to 38 g liter^–1^ salinity and 25°C temperature. Light intensity was set to 170 μmol photons m^–2^ s^–1^ with a 12 h light/12 h dark photoperiod with HQI10000K metal halide lamps (BLV Nepturion). Total alkalinity (TA) and pH were measured daily, and carbonate seawater chemistry was calculated as described previously (Tambutté et al., 2015).

### DNA and RNA Extraction, Reverse Transcription, and 16S rRNA Gene and Transcript Amplicon Sequencing

*Stylophora pistillata* DNA was extracted with DNeasy PowerBiofilm (Qiagen, Hilden, Germany) following manufacturer’s instructions. Briefly, small pieces from nubbins of *S. pistillata* were collected in PowerBiofilm Bead tubes and homogenized with CryoMill (Retsch, Germany). Triplicates were extracted for each pH condition. Following centrifugation, supernatants were passed through Silica Spin Filter Tubes and after washing steps, DNA was eluted in 10 mM Tris.

Total RNA was isolated from triplicates collected for each pH treatments using RNeasy kit (Qiagen) according to manufacturer’s instructions. First-strand cDNA was synthesized using a SuperScript III First Strand Synthesis SuperMix Kit (Invitrogen). Reactions were done using a mixture of annealing buffer and random hexamers, with 20 μl total volume. Thermo cycler conditions were as follows: 50 min at 50°C, 7 min at 25°C, 50 min at 50°C and 5 min at 85°C.

For 16S rRNA amplicon sequencing, triplicate PCR reactions of 25 μl final volume were done using the Qiagen Multiplex PCR kit using 30 ng/μl of template DNA and cDNA. For amplification we targeted variable regions 5 and 6 of the 16S rRNA gene using primers 784F (5′TCGTCGGCAGCGTCAGATGTGTATAAGAGACAGAGGA TTAGATACCCTGGTA′3) and 1061R (5′GTCTCGTGGGC TCGGAGATGTGTATAAGAGACAG-CRRCACGAGCTGACG AC′3) (Andersson et al., 2008; Bayer et al., 2013) with Illumina adapter overhangs (underlined). Final primer concentration was of 0.5 μM. PCR conditions consisted of initial denaturing step at 95°C for 15 min, followed by 27 cycles of 95°C for 30 s, 55°C for 90 s, and 72°C for 30 s, and a final extension step at 72°C for 10 min. To confirm amplification from the right size, we ran 1% agarose gel using 4 μl PCR product.

### Library Preparation and Sequencing

Triplicate PCRs for each sample were pooled, and cleaned using the Mini Elute PCR purification kit (Qiagen, Hilden, Germany), following manufacturer instructions. PCR products were indexed with Nextera XT barcoded sequencing adapters (Illumina, San Diego, CA, United States) and cleaned again following manufacturer instructions. Indexed PCR products were then quantified with QuBit (dsDNA High Sensitivity Assay Kit, Thermo Fisher Scientific, Waltham, MA, United States) and pooled in equimolar ratios. Pooled samples were inspected for quality on the BioAnalyzer (Agilent Technologies, Santa Clara, CA, United States) and sequenced at 6 pM on the Illumina MiSeq, 2 bp × 300 bp paired-end v3 chemistry according to the manufacturer instructions.

### Sequence Data Processing and Analysis

Raw reads were quality-filtered (QV = 25) and decontaminated of Illumina adapter and phiX sequences using the BBDuk tool from the BBMap v37.62 suite (Bushnell B^1^). Mothur v1.44.3 (Schloss et al., 2009) was used for sequence data processing and analysis. Briefly, forward and reverse reads were assembled into contigs and ambiguous reads removed before quality trimming and pre-clustering took place (Huse et al., 2010). After exclusion of singletons, sequences were aligned to the Silva database 138.1 (Pruesse et al., 2007). Chimeric sequences were further excluded with UCHIME (Edgar et al., 2011), as were chloroplast, mitochondrial, archaeal, and eukaryotic sequences using the remove.lineages command in mothur. Sequences were then classified against the Silva database (McDonald et al., 2012). Sequences were reclassified against the Silva database after subsampling to 10,669 sequences, which was the highest number of sequences possible while still keeping all replicates. Operational Taxonomic Units (OTUs) were defined based on sequence clustering at a 97% similarity cutoff and used for following analysis.

We used R (version 3.5.1, 2018) and PRIMER E v6 (PERMANOVA+) software for multivariate analysis on Log (x + 1) transformed OTU count data (Clarke and Gorley, 2006). Difference between groups according to genetic material (i.e., DNA vs. RNA) and pH treatment were visualized with non-metric MultiDimensional Scaling (nMDS) plots, based on Bray-Curtis dissimilarity distances. One-factorial PERmutational MANOVAs (PERMANOVAs) were used to test for differences between DNA vs. RNA material and pH treatments. We also performed an analysis of similarity percentages (SIMPER; Clarke, 1993) based on Bray Curtis resemblance matrix to identify OTUs mainly responsible for dissimilarity between groups. Alpha diversity indices (Chao1, Inverse Simpson, Simpson Evenness) were calculated for each sample in mothur. Samples were tested for normality and homoscedasticity with Shapiro–Wilk and Levene’s test, before being analyzed for differences between pH treatments using ANOVA with Tukey *post hoc* tests in R (version 3.5.1, 2018). The putative core microbiome of *S. pistillata* was defined by identifying bacterial OTUs present in all treatment and replicates.

Significantly differences in OTU abundance amongst treatments were analyzed by the linear discriminant analysis (LDA) effect size (LEfSe) method (Segata et al., 2011) on the website https://huttenhower.sph.harvard.edu/galaxy/root (LDA > 2).

## Results

### Sample and Sequencing Overview

Illumina MiSeq sequencing of bacterial 16S rDNA and rRNA amplicons generated a total of 4,157,907 sequences from 24 samples [average 173,247 ± 100,095 (SD)], being 1,482,981 for RNA samples and 2,674,938 for DNA samples (Supplementary Table 1). After quality filtering and exclusion of chimeras and unwanted sequences, 1,474,649 remained, with an average of 61,444 ± 38,313 (SD). From these, 14,829 distinct sequences were identified after clustering at 97% similarity and assigned to bacteria. Subsampling was done to 10,669 sequences, which was the highest number that allowed keeping all replicates. After quality filtering, DNA samples contained an average of 76,488 sequences (range from 13,735 to 165,630), while RNA samples contained an average of 46,399 sequences (range from 10,669 to 114,558, Supplementary Table 1). Rarefaction curves for DNA and RNA samples suggested that subsampling to 10,669 was indeed sufficient to capture most of bacterial diversity (Supplementary Figure 1A).

### Distinct Bacterial Communities Based on 16S rDNA and 16S rRNA Sequencing

**FIGURE 1:**
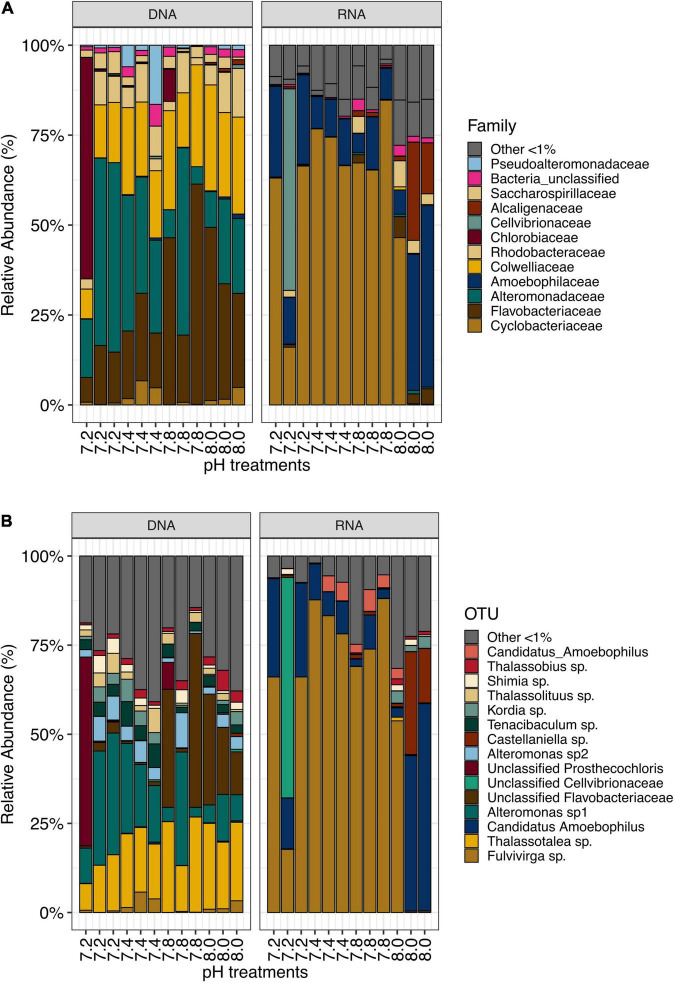
**(A)** Most abundant bacterial families (>1%) and **(B)** OTUs in samples from *Stylophora pistillata* kept in aquaria for 2 years at different pH treatments (pH 7.2, 7.4, 7.8 and 8.0), after subsampling to 10,669 reads.

We identified a total of 154 bacterial families for DNA- and 89 bacterial families for RNA-based sequencing. DNA samples were dominated by members of Alteromonadaceae (24.52%), Flavobacteriaceae (24.17%) and Colwelliaceae (18.51%), while RNA samples were dominated by families Cyclobacteriacea and Amoebophilaceae (57.97 and 20.69%, respectively, Figure 1A). For further analyses, sequences were clustered into Operational Taxonomic Units (OTUs) at a 97% similarity level. A total of 3,266 OTUs were identified from this dataset. DNA samples contained 2,047 OTUs, while RNA samples contained 1,659 OTUs. DNA samples were dominated by *Thalassotalea* sp. (18.37% relative abundance, OTU0002, 100% identical to *Thalassotalea* sp. GenBank accession no. MN822801.1), *Alteromonas* sp. (16.54% relative abundance, OTU0003, 100% identical to *Alteromonas macleodii*, GenBank accession no. CP018321.1) and unclassified Flavobacteriaceae (12.77% relative abundance, OTU0005, 98.95% identical to *Polaribacter* sp., GenBank accession no. MK818916.1, Figure 1B). In contrast, *Fulvivirga* sp. (OTU0001, 95.85% identical to *Fulvivirga* sp., GenBank accession no. JQ516517.1) dominated RNA samples (57.00% relative abundance), followed by *Candidatus amoebophilus*, (OTU0004, 17.69%, Figure 1B).

**FIGURE 2:**
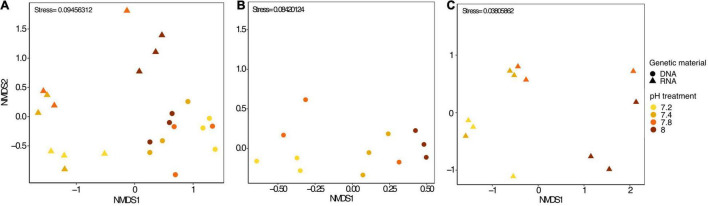
**(A)** nMDS plot including all microbial samples (DNA and RNA), **(B)** only microbial DNA samples and **(C)** only microbial RNA samples from *Stylophora pistillata* kept in aquaria for 2 years at different pH treatments (pH 7.2, 7.4, 7.8 and 8.0). Data was transformed to Log (x + 1) and resemblance matrix based on Bray Curtis similarity.

In each pH treatments, some OTUs were present exclusively in RNA samples while others were exclusively found in DNA samples (Supplementary Figures 1B–E). Yet, most of the top abundant OTUs were present in both DNA and RNA samples, (Supplementary Table 2) despite a noticeable disparity in abundance. For instance, the most abundant bacterial OTU found in DNA, *Thalassotalea* sp., (OTU0002), only accounted for 0.18% in RNA samples. Similarly, *Fulvivirga* sp. (OTU0001) accounted for more than half of the bacterial community in RNA samples, yet, they only represented 1.47% of the relative abundance in DNA samples. The next three most abundant taxa found in DNA samples accounted for only between 0.007 and 0.04% of the relative abundance in RNA samples. Similarly, following the same pattern, the next three most abundant taxa in RNA samples accounted for only between 0.15 and 0.17% of the relative abundance in DNA samples (Supplementary Figure 2A). In fact, *Stylophora pistillata’s* bacterial community was distinct when comparing 16S rDNA and 16S rRNA sequencing results from the same samples (PERMANOVA, pMC = 0.0001, Figure 2A). Furthermore, Simper analysis indicated a dissimilarity value between DNA and RNA samples of 87.14% (Supplementary Table 3). *Alteromonas* sp. (OTU0004), *Thalassotalea* sp. (OTU0002) and *Tenacibaculum* sp. (OTU0010) contributed the most to the DNA-RNA dissimilarity (Supplementary Table 4).

### Decreased pH Has Larger Impact on Active Bacterial Communities

**FIGURE 3:**
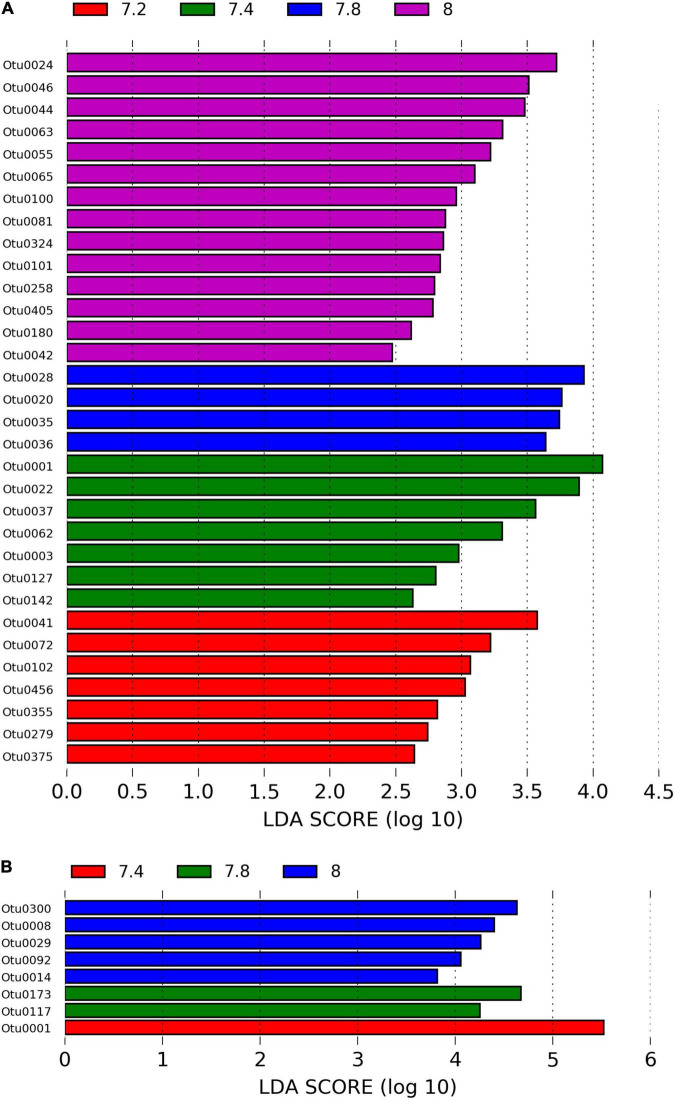
**(A)** LEfSe analysis identifying representative OTUs from microbial DNA samples and **(B)** microbial RNA from *Stylophora pistillata* kept in aquaria for 2 years at different pH treatments (pH 7.2, 7.4, 7.8 and 8.0), with a cutoff value of LDA score (log_10_) above 2.

We identified approximately even numbers of OTUs in DNA samples with a total of 730 OTUs at pH 8.0, 717 at pH 7.8, 796 at 7.4, and 871 at 7.2 in the DNA samples and 131 OTUs were shared between all pH groups (Supplementary Figure 2B). For RNA samples, the number of OTUs was decreasing with pH, with 1,078 bacterial OTUs at pH 8.0, 759 at pH 7.8, 166 at pH 7.4 and 169 at pH 7.2, with only 33 shared OTUs between all pH groups (Supplementary Figure 2C). When considering only the DNA data, there was a significant general effect of pH (PERMANOVA, pMC = 0.02, Pseudo *F* = 2.0182), with significant differences between pH 7.2 and 8.0 (pMC = 0.0312, *t* = 1.9779, Figure 2B). We observed increased abundance of families Flavobacteriaceae at pH 7.8 and 8.0 (40 and 29%), and Chlorobiaceae and Alteromonadaceae at pH 7.2 (19 and 37% respectively, Figure 1A). At OTU level, unclassified Flavobacteriaceae (OTU0005) was relatively more abundant at pH 7.8 and 8.0 (28 and 21%, respectively), while *Alteromonas* sp. (OTU0004) and *Prostheocochloris* sp. (OTU0007) was more abundant at 7.2 pH treatments (25 and 18%, respectively) (Figure 1B). LEfSe analysis indicated 14 representative OTUs for pH 8.0, four for pH 7.8, seven for pH 7.4 and seven for pH 7.2 in DNA samples (Figure 3A).

When considering only the RNA data, there was also a significant effect of pH (PERMANOVA, pMC = 0.0044, Pseudo *F* = 2.8021, Figure 2C), yet with significant differences between pH 7.2 and 8.0 (pMC = 0.0332, *t* = 1.9342) and between pH 7.4 and 8.0 (pMC = 0.0228, *t* = 2.1838). Cellvibrionaceae was more abundant at pH 7.2, while Cyclobacteriaceae decreased in relative abundance at pH 8.0. Families Alcaliginaceae, Amoebophilaceae, Rhodobacteriaceae and Flavobacteriaceae were more abundant at pH 8.0 (Figure 1A). At OTU level, *Castellaniella* sp. and “*Candidatus Amoebophilus”* were more abundant at pH 8.0. *Fulvivirga* sp. was less abundant at pH 8.0, while unclassified Cellvibrionaceae was more abundant at pH 7.2 (Figure 1B). LEfSe analysis identified five microbial OTUs enriched at pH 8.0, two at pH 7.8 and one enriched at pH 7.4 (Figure 3B).

Alpha diversity measures (Chao estimate of species richness, Shannon diversity and Simpson evenness indices) were calculated using mothur. While Shannon diversity index was significantly higher in the control treatment (pH 8.0) compared to 7.2 and 7.4 pH (ANOVA, *p* < 0.05; Tukey, *p* < 0.05) at the RNA level, the same was not observed at the DNA level (Table 1). Although the control treatment showed a pattern of higher Chao estimated richness at RNA level than at low pH, and higher in pH 7.2 at DNA level than at high pH, this was not statistically significant (Kruskal–Wallis, *p* > 0.05). Community evenness was similar amongst pH treatments at DNA level (ANOVA, *p* < 0.05), while a significant decreasing pattern was observed from low to high pH at RNA level with the least even communities at control pH (ANOVA, *p* < 0.05, Table 1).

**TABLE 1:** Differences in alpha diversity indices (Chao estimate of species richness, Shannon and Simpson evenness) in microbiome of *Stylophora pistillata* kept in aquaria for 2 years at different pH treatments (7.2; 7.4, 6.8 and 8.0) for DNA and RNA samples.

Treatment		Chao1			Shannon			Simpson evenness		
		Mean	SD	SE	Mean	SD	SE	Mean	SD	SE
DNA	7.2	765.94	100.73	58.16	2.83	0.35	0.20	0.01	0.00	0.00
	7.4	494.74	323.31	186.66	3.30	0.25	0.15	0.04	0.02	0.01
	7.8	492.58	260.52	150.41	2.54	0.66	0.38	0.02	0.02	0.00
	8	410.36	142.16	82.08	3.38	0.43	0.25	0.03	0.00	0.00
RNA	7.2	147.16	45.09	26.03	1.08	0.14	0.08	0.03	0.00	0.00
	7.4	164.47	59.28	34.22	0.74	0.23	0.14	0.02	0.01	0.00
	7.8	492.65	552.17	318.79	1.34	0.82	0.47	0.01	0.01	0.00
	8	684.66	130.10	75.11	2.42	0.43	0.25	0.01	0.00	0.00

### Core Microbiome

Bacterial OTUs that were present in all treatments and all replicates (from DNA samples only) were considered to constitute the putative core microbiome. This resulted in 23 taxa that were consistently found to be part of the microbiome of *Stylophora pistillata* (Table 2). Amongst them, *Thalassotalea* sp. (18.37% of relative abundance), *Alteromonas* sp. (16.54%) and unclassified Flavobacteriaceae (12.77%) were the most abundant taxa. The remaining taxa showed substantially lower relative abundances, ranging from 4.18 to 0.04%.

**TABLE 2:** Core microbiome of *Stylophora pistillata* kept in aquaria for 2 years at different pH treatments (7.2; 7.4, 7.8 and 8.0).

OTU	Taxon	Average Abundance				Relative abundance%	Blast on NCBI database		
		7.2	7.4	7.8	8		Total score	Query cover	Identity
Otu0002	Thalassotalea	1,295.7	1,927.3	2,312.3	2,304.0	18.37	536	98%	*Thalassotalea* sp.
Otu0004	Alteromonas	2,703.7	2,082.0	1,368.7	904.3	16.54	536	100%	*Alteromonas marina*
Otu0005	Flavobacteriacea	218.0	81.7	2,946.3	2,202.3	12.77	527	100%	*Flexibacter* sp.
Otu0008	Alteromonas	558.7	481.0	420.3	325.7	4.18	536	98%	*Alteromonas* sp.
Otu0010	Tenacibaculum	354.7	626.0	271.0	271.7	3.57	525	100%	*Tenacibaculum holothuriorum*
Otu0012	Thalassolituus	401.3	368.7	224.7	149.7	2.68	525	98%	*Thalassolituus oleivorans*
Otu0011	Kordia	296.7	481.7	23.0	189.7	2.32	503	100%	*Kordia* sp.
Otu0013	Shimia	375.0	182.3	171.7	132.3	2.02	531	98%	*Shimia* sp.
Otu0014	Thalassobius	111.7	177.7	156.7	395.7	1.97	534	98%	*Mameliella alba*
Otu0001	Fulvivirga	40.3	390.3	15.3	188.0	1.49	462	100%	*Fulvivirga* sp.
Otu0018	Salinimonas	133.7	92.7	103.0	101.3	1.01	536	98%	*Alteromonas* sp.
Otu0020	Tenacibaculum	12.7	47.7	177.0	140.3	0.88	496	100%	*Tenacibaculum holothuriorum*
Otu0019	Unclassified bacteria	14.0	247.7	6.0	65.7	0.78	357	98%	*Leptospira ryugenii*
Otu0023	Oleiphilus	64.7	82.7	64.0	93.3	0.71	492	100%	*Oleiphilus* sp.
Otu0024	Pelomonas	10.3	125.7	12.0	152.7	0.70	538	98%	*Pelomonas aquatica*
Otu0025	Cutibacterium	19.3	75.0	79.0	127.0	0.70	542	100%	*Rhodococcus* sp.
Otu0027	Pseudoalteromonas	16.7	254.3	8.0	4.3	0.66	529	98%	*Pseudoalteromonas* sp.
Otu0028	Flavobacteriaceae	15.7	3.7	202.0	55.3	0.65	510	100%	*Polaribacter* sp.
Otu0031	Endozoicomonas	7.7	119.7	3.7	61.3	0.45	510	94%	*Endozoicomonas* sp.
Otu0032	Vibrio	27.0	54.0	70.0	25.7	0.41	536	98%	*Vibrio alginolyticus*
Otu0039	Microscilla	49.0	10.0	24.0	15.7	0.23	529	100%	*Microscilla*
Otu0043	Ruegeria	9.7	33.0	22.0	16.3	0.19	534	98%	*Ruegeria* sp.
Otu0095	Rhodobacteraceae	4.7	5.3	4.3	4.3	0.04	529	98%	*Sulfitobacter* sp.

*Only DNA samples were considered when defining the core microbiome.*

## Discussion

### Discrepancy Between Resident and Active Bacterial Communities in Response to Ocean Acidification

This is the first research to assess the effects of OA on a coral microbiome considering both the resident and active bacterial communities. Sequencing of 16S rDNA gene is a *de facto* standard method to identify microbial communities. However, a major disadvantage of this method is that DNA can remain in the environment after cell death, making it challenging to distinguish active from dormant populations or even extracellular material (Carini et al., 2016). Since RNA is short-lived, and ribosome quantity is generally associated with active growth and cellular activity, some authors consider sequencing of reverse transcribed rRNA a more accurate representation of metabolically active microbial communities (Ramos et al., 2000).

In this study, we found differences in bacterial community composition and relative abundance when sequencing the resident (DNA) and active (RNA) communities from the same samples. The top OTUs most contributing to these differences were around 4–8 times more abundant in the resident than the active community. Interestingly, a previous study on marine sediments detected 16S rDNA gene abundances of bacterial communities 1–2 orders of magnitude higher than the transcript abundances (Li et al., 2020). Conversely, some OTUs were more abundant in the active community. This decoupling could be explained by either a response to specific treatment or by a substantial fraction of bacteria becoming active when their abundance decreased. This could suggest that high abundance limits activity or that top-down processes, such as grazing and virus lysis, could be involved in promoting or regulating activity (Berdjeb et al., 2011).

Interestingly, we found many OTUs exclusively in either the resident or the active communities, with only 13.5% overlap between both sources. However, the majority of the most abundant OTUs were present in both fractions. Previous studies found that only 29–50% of 16S rDNA sequences were identical to sequences in the 16S rRNA library from the same soil (Nogales et al., 2001; Mengoni et al., 2005; Lillis et al., 2009). Other studies also detected stark differences between 16S rDNA and rRNA libraries from soil samples (Baldrian et al., 2012; Romanowicz et al., 2016) and the coral *L. pertusa* (Galand et al., 2018). Undetected OTUs may reflect low abundance below PCR amplification levels (Mengoni et al., 2005) or they could be excluded during subsampling. In a biological context, the presence of a specific taxa in only rDNA samples could indicate lack of activity, while exclusively detecting taxa on the rRNA level might indicate rare species that are highly active. It could also indicate colonization by opportunistic bacteria that are likely not active and have low ecological relevance (Galand et al., 2018).

Our results showed higher OTU richness and diversity for resident compared to active communities, indicating that the metabolically active bacterial components in *S. pistillata* constitute only a fraction of the total microbiome. A similar pattern was observed by other authors for bacterial populations in the rhizosphere of Chrysanthemum (Duineveld et al., 2001), coral reef and marine sediments (Gaidos et al., 2011), as well as other studies based on sequencing of bacterial communities from different environments (Lanzén et al., 2011; Weigold et al., 2016). The higher richness and diversity of DNA based libraries is likely a reflection of recovering a broader range of microbes, including active, dormant and even dead cells as opposed to only active cells detected by RNA based libraries (Salgar-Chaparro and Machuca, 2019).

However, it is important to note that bias introduced by PCR-induced artifacts and different extraction methods for DNA and RNA, including the use of RNA protection agents, is a recurrent problem when characterizing microbial communities (Acinas et al., 2005; McCarthy et al., 2015).

### Ocean Acidification Effects Are Distinct Between Resident and Active Microbial Communities

Many studies have evaluated the effects of ocean acidification on the coral microbiome, but most of them focused only on sequencing of 16S rDNA (O’Brien et al., 2016). While these studies found some effect of OA on community composition and function (Webster et al., 2013, 2016; Morrow et al., 2015; Grottoli et al., 2018), others reported a stable microbiome (Meron et al., 2012; Zhou et al., 2016; O’Brien et al., 2018). In this study, we detected a significant difference in bacterial communities from pH 7.2 and pH 8.0 in both active and resident bacterial communities, and from pH 7.4 and 8.0 in the active community. Overall, we found stronger differences in response to OA in the active compared to the resident bacterial communities. A similar pattern was observed by other authors in response to pollution and toxic compounds (Mengoni et al., 2005; Hoshino and Matsumoto, 2007; Lillis et al., 2009). This pattern is, to some extent, expected since the resident community also encompasses dead and non-active cells that will not respond to changes in the environment. Thus, our data support the notion that RNA better describes the actively responding community, which should show the most direct effects of disturbance (Mengoni et al., 2005).

We observed a drop in richness and diversity in the active community with decreasing pH, suggesting growth and activity limitation at low pH for many bacterial OTUs. For instance, *Castellaniella* sp. (OTU 0009, 100% identical to *Castellaniella denitrificans*, GenBank accession no. MK039104.1), which is from the Alcaligenaceae family and known to be involved in denitrification processes (Rosenberg, 2013) was present in the resident community at all pH levels, but decreased with pH in the active community. Similarly, studies on denitrifying prokaryotes in soil, wastewater sludges and batch reactor cultures found a decrease in denitrification activity in low pH (<6.5) (Thörn and Sörensson, 1996; Ellis et al., 1998). Denitrifying prokaryotes are found in coral holobionts worldwide (Gaidos et al., 2011; Yang et al., 2013; Rädecker et al., 2015; Tilstra et al., 2019). Denitrification by members of the coral microbiome may have a potential role in contributing to a balanced nitrogen to phosphorous ratio by decreasing nitrogen levels in coral tissues. Maintaining a N-limited state may generally contribute to holobiont functioning and health through the regulation of Symbiodiniaceae proliferation (Rädecker et al., 2015; Cui et al., 2019; Tilstra et al., 2019). In fact, previous studies using the same coral colonies did not observe a decrease in symbiont density with pH decrease (Tambutté et al., 2015). Consequently, a decrease in denitrifying bacteria at lower pH treatments could potentially impact nitrogen balance in coral holobionts.

Our LEfSe analysis indicated that *Alteromonas* sp. (OTU0008) and *Lacimicrobium* sp. (OTU0029) were representatives of the active community under control conditions at pH 8.0, and nearly absent in lower pH treatments. Many species of the family Alteromonadaceae, especially *Alteromonas*, are sulfate reducers and known to be involved in DMSP metabolism (Raina et al., 2009), which is linked to osmoregulation and antioxidant capacity (Gardner et al., 2016; Hopkins et al., 2016). Previous studies also associated this genus with disease resistance (Wright et al., 2017). Thus, the decrease in abundance of members of this genus at low pH treatments could indicate loss in resilience and biogeochemical functions. Nonetheless, the functional role and ecological relevance of many bacterial taxa is still unknown, and this research area is essential for understanding holobiont functioning during stress. Even though OA can negatively influence microbial communities, the implication of those shifts on the host health, ecosystem function and adaptation or acclimation mechanisms are still being investigated. In contrast, we found that OA stimulated growth and activity of specific OTUs, such as unclassified Cellvibrionaceae (OTU0006, 98.28% identical to *Exilibacterium tricleocarpae*, GenBank accession no. MN094885.1). This OTU was present in low abundance in the resident community, suggesting that it might represent an opportunistic bacterium that increased in activity in response to acidification in low pH treatments.

Interestingly, richness and diversity in the resident community did not differ statistically between treatments, and remained high even in low pH treatments, suggesting that many bacterial taxa remain in the community after environmental change as dormant members. Dormancy plays an important role in shaping bacterial communities and is a well-known strategy used by many organisms to survive through unfavorable conditions (Sussman and Douthit, 1973). Previous studies indicate that dormant bacteria can account for high proportion of taxa in stressed ecosystems (Jones and Lennon, 2010). Some resident representative OTUs identified by LEfSe analysis at pH 7.2 were members of the phylum Bacteroidetes, including the genus *Tenacibaculum* (OTU0279), a known fish pathogen (Faílde et al., 2013) and members of the Flavobacteriaceae family (OTU0041 and OTU0375). A higher abundance of Bacteroidetes has been reported previously for corals under low pH conditions (Thurber et al., 2009; Meron et al., 2011; Morrow et al., 2015) and in diseased coral (Bourne et al., 2009). Similarly, three indicator species for pH 7.2 were from the order Alteromonadales, which encompass some potential coral pathogens (Krediet et al., 2013; Roder et al., 2014).

Thus, a change in OA can induce shifts in the bacterial community by inhibiting activity of many taxa while stimulating activity of a few specific, potentially opportunistic, taxa previously resident in low abundances. Despite the identified changes on microbial community composition in response to OA, the implications for host health are still unknown. In fact, changes in the coral microbiome due to environmental stress do not necessarily reflect holobiont stress, but could even reflect potential acclimation responses. Thus, more studies should be conducted to investigate consistent patterns in microbiome changes associated with environmental stressors, as well as acclimation potential and ecological functions of key microbial taxa in coral phenotypes.

### Core Microbiome of *Stylophora pistillata*

To study patterns in microbiome composition, previous studies have focused on the core microbiome, which is defined as the members consistently shared among microbial consortia from similar habitats (Shade and Handelsman, 2012). Usually, the coral microbiome has a core species-specific bacterial group and a transient group that changes according to environmental parameters such as climate and geography (Ritchie, 2006).

*S. pistillata* has previously been shown to have specific associations with species from the genus *Endozoicomonas* (Bayer et al., 2013; Neave et al., 2017) and we also identify them as part of the core microbiome in this study (Table 2). However, their abundance is lower than reported in studies using colonies sampled from the wild, potentially due to the long-term rearing of the colonies used here in an aquarium environment. Coral microbiome studies greatly depend on corals maintained in aquariums that simulate natural conditions, but the effects of captivity on the coral microbiome are understudied. Previous research has observed differences in the microbiome of corals between colonies *in situ* and those reared in aquaria. For instance, shifts on the surface mucus layer (SML) microbiome were reported for *Fungia granulosa* (Kooperman et al., 2007) and *Siderastrea siderea* after as early as 1 day of aquarium rearing (Pratte et al., 2015). In the coral *Lophelia pertusa*, both skeleton and SML were different between *in situ* and *ex situ* habitats (Schöttner et al., 2009). In *Eguchipsammia fistula*, the associated microbiome also differed between native and aquarium settings. Functional profiling suggested that processes related to carbon-and nitrogen-limited environments were enriched in the native habitat, which could reflect diet changes between *in situ* and aquarium conditions (Röthig et al., 2017). Galand et al. (2018) investigated microbiome changes in aquaria across different time points, both on the active (rRNA) and resident (rDNA) fraction. Their results suggest a species-specific response. *For Madrepora oculata*, bacterial associations remained stable for at least 6 months of captivity, which is in agreement with a stable bacterial community in aquarium-reared *Acropora loripes* over 4 weeks (Hill and Ralph, 2006; Damjanovic et al., 2020). Interestingly, Galand et al. (2018) further found that the abundance of *Endozoicomonas* in the RNA fraction decreased after only 1 day of captivity but started to increase again after 5 days. For *Lophelia pertusa*, they observed microbiome changes after only 1 day of aquarium rearing. Some taxa disappeared completely and other appeared only after months in aquaria, which the authors considered an aquarium signature. They further concluded that opportunistic bacteria might colonize corals in captivity. Thus, considering the long-term nature of this study’s experimental setup, with corals kept in captivity for several decades, it is possible that their microbiomes diverged significantly from the ones found in natural environments. This illustrates a potential limitation of using aquarium specimens for microbial community studies, since it will limit the presence of potentially ecological relevant bacterial taxa associated to geographical location.

### Importance of the Active Microbiome

In this study, we identified bacterial taxa with low relative abundance on DNA level, but high abundance on RNA level, indicating rare but highly active community members. The existence of a large diverse microbial community, with few abundant and many rare taxa is common in the natural environment (Sogin et al., 2006). While the relationship between bacterial abundance and activity is still unclear, one common hypothesis for this distribution is that abundant bacteria have high growth rates, while rare taxa are slow growing (Pedrós-Alió, 2006). However, multiple studies have found rare bacteria taxa to be disproportionally active and displaying high growth rates (Jones and Lennon, 2010; Campbell et al., 2011; Weigold et al., 2016).

There is an increasing number of studies highlighting the importance of rare bacterial taxa (Sogin et al., 2006; Pedrós-Alió, 2007, 2012; D Ainsworth et al., 2015; Lynch and Neufeld, 2015; Jousset et al., 2017). These often underrated and unexplored species may represent a source of genetic novelty in times of environmental change. Some might even be keystone species, being essential to understand community composition and function (Sogin et al., 2006; Elshahed et al., 2008).

## Conclusion

The use of 16S rDNA sequencing to study bacterial communities is widespread. However, only few studies use 16S rRNA sequencing, which means much is unknown about the correlation between microbial activity and composition, particularly for coral holobionts. We show that OA can significantly impact the resident coral microbiome, although a stronger effect was observed on the active microbial community, which suggests that the microbial community responded primarily to the environmental change through modified activity patterns. Importantly, we also found that low pH might play a role in the shift of bacterial OTUs from active to dormant states and vice versa, allowing opportunistic bacteria to increase in activity while reducing the abundance of some members of the core microbiome. The microbiome is a critical component of coral resilience, and this study provides insights into the effects of ocean acidification on coral bacterial community composition and coral holobiont response to climate change. Moreover, our study confirms that rDNA and rRNA based sequencing can result in different community composition, with the RNA-based community reflecting the metabolically active population. Our analysis also showed that some low abundant resident bacterial taxa were highly active, suggesting they could play key functional roles. Thus, both methods, rDNA and rRNA sequencing, should be considered complementary tools to investigate microbial assemblage structure and activity.

## Data Availability Statement

The datasets presented in this study can be found in online repositories. The names of the repository/repositories and accession number(s) can be found below: https://www.ebi.ac.uk/ena, PRJEB44699.

## Author Contributions

DZ, ST, DA, ET, and AV performed the experiment in Monaco. MZ, CV, and MA designed the study. MB performed laboratory work, analyzed the data, and wrote the first draft of the manuscript. CA contributed to the bioinformatics analysis. All authors contributed to the writing and editing of the manuscript and read and approved the final manuscript.

## Conflict of Interest

The authors declare that the research was conducted in the absence of any commercial or financial relationships that could be construed as a potential conflict of interest.

## Publisher’s Note

All claims expressed in this article are solely those of the authors and do not necessarily represent those of their affiliated organizations, or those of the publisher, the editors and the reviewers. Any product that may be evaluated in this article, or claim that may be made by its manufacturer, is not guaranteed or endorsed by the publisher.

## Footnotes

1
http://sourceforge.net/projects/bbmap/

## References

[B1] Acinas S. G. Sarma-Rupavtarm R. Klepac-Ceraj V. Polz M. F. (2005). PCR-induced sequence artifacts and bias: insights from comparison of two 16S rRNA clone libraries constructed from the same sample. *Appl. Environ. Microbiol.* 71 8966–8969. 10.1128/AEM.71.12.8966-8969.2005 16332901 PMC1317340

[B2] Andersson A. F. Lindberg M. Jakobsson H. Bäckhed F. Nyrén P. Engstrand L. (2008). Comparative analysis of human gut microbiota by barcoded pyrosequencing. *PLoS One* 3:e2836. 10.1371/journal.pone.0002836 18665274 PMC2475661

[B3] Anthony K. R. N. Kline D. I. Diaz-Pulido G. Dove S. Hoegh-Guldberg O. (2008). Ocean acidification causes bleaching and productivity loss in coral reef builders. *Proc. Natl. Acad. Sci. U.S.A.* 105 17442–17446. 10.1073/pnas.0804478105 18988740 PMC2580748

[B4] Baldrian P. Kolařík M. Stursová M. Kopecký J. Valášková V. Větrovský T. (2012). Active and total microbial communities in forest soil are largely different and highly stratified during decomposition. *ISME J.* 6 248–258. 10.1038/ismej.2011.95 21776033 PMC3260513

[B5] Bayer T. Neave M. J. Alsheikh-Hussain A. Aranda M. Yum L. K. Mincer T. (2013). The microbiome of the red sea coral stylophora pistillata is dominated by tissue-associated endozoicomonas bacteria. *Appl. Environ. Microbiol.* 79 4759–4762. 10.1128/AEM.00695-13 23709513 PMC3719505

[B6] Berdjeb L. Pollet T. Domaizon I. Jacquet S. (2011). Effect of grazers and viruses on bacterial community structure and production in two contrasting trophic lakes. *BMC Microbiol.* 11:88. 10.1186/1471-2180-11-88 21527043 PMC3114703

[B7] Blagodatskaya E. Kuzyakov Y. (2013). Active microorganisms in soil: critical review of estimation criteria and approaches. *Soil Biol. Biochem.* 67 192–211. 10.1016/J.SOILBIO.2013.08.024

[B8] Blazewicz S. J. Barnard R. L. Daly R. A. Firestone M. K. (2013). Evaluating rRNA as an indicator of microbial activity in environmental communities: limitations and uses. *ISME J.* 7 2061–2068. 10.1038/ismej.2013.102 23823491 PMC3806256

[B9] Bourne D. G. Garren M. Work T. M. Rosenberg E. Smith G. W. Harvell C. D. (2009). Microbial disease and the coral holobiont. *Trends Microbiol.* 17 554–562. 10.1016/J.TIM.2009.09.004 19822428

[B10] Campbell B. J. Yu L. Heidelberg J. F. Kirchman D. L. (2011). Activity of abundant and rare bacteria in a coastal ocean. *Proc. Natl. Acad. Sci. U.S.A.* 108 12776–12781. 10.1073/pnas.1101405108 21768380 PMC3150899

[B11] Carini P. Marsden P. J. Leff J. W. Morgan E. E. Strickland M. S. Fierer N. (2016). Relic DNA is abundant in soil and obscures estimates of soil microbial diversity. *Nat. Microbiol.* 2 1–6. 10.1038/nmicrobiol.2016.242 27991881

[B12] Clarke K. R. (1993). Non-parametric multivariate analyses of changes in community structure. *Austral. Ecol.* 18 117–143. 10.1111/j.1442-9993.1993.tb00438.x

[B13] Clarke K. R. Gorley R. N. (2006). *PRIMER V6: User Manual/Tutorial.* Plymouth: PRIMER-E.

[B14] Cui G. Liew Y. J. Li Y. Kharbatia N. Zahran N. I. Emwas A.-H. (2019). Host-dependent nitrogen recycling as a mechanism of symbiont control in *Aiptasia*. *PLoS Genet.* 15:e1008189. 10.1371/journal.pgen.1008189 31233506 PMC6611638

[B15] D Ainsworth T. Krause L. Bridge T. Torda G. Raina J.-B. Zakrzewski M. (2015). The coral core microbiome identifies rare bacterial taxa as ubiquitous endosymbionts. *ISME J.* 9 2261–2274. 10.1038/ismej.2015.39 25885563 PMC4579478

[B16] Damjanovic K. Blackall L. L. Peplow L. M. van Oppen M. J. H. (2020). Assessment of bacterial community composition within and among Acropora loripes colonies in the wild and in captivity. *Coral Reefs* 39 1245–1255. 10.1007/s00338-020-01958-y

[B17] DeAngelis K. M. Firestone M. K. (2012). Phylogenetic clustering of soil microbial communities by 16S rRNA but not 16S rRNA genes. *Appl. Environ. Microbiol.* 78 2459–2461. 10.1128/AEM.07547-11 22286992 PMC3302588

[B18] Dlott G. Maul J. E. Buyer J. Yarwood S. (2015). Microbial rRNA: RDNA gene ratios may be unexpectedly low due to extracellular DNA preservation in soils. *J. Microbiol. Methods* 115 112–120. 10.1016/j.mimet.2015.05.027 26055315

[B19] Doney S. C. Fabry V. J. Feely R. A. Kleypas J. A. (2009). Ocean acidification: the other CO 2 problem. *Ann. Rev. Mar. Sci.* 1 169–192. 10.1146/annurev.marine.010908.163834 21141034

[B20] Duineveld B. M. Kowalchuk G. A. Keijzer A. van Elsas J. D. van Veen J. A. (2001). Analysis of bacterial communities in the rhizosphere of chrysanthemum via denaturing gradient gel electrophoresis of PCR-amplified 16S rRNA as well as DNA fragments coding for 16S rRNA. *Appl. Environ. Microbiol.* 67 172–178. 10.1128/AEM.67.1.172-178.2001 11133442 PMC92540

[B21] Edgar R. C. Haas B. J. Clemente J. C. Quince C. Knight R. (2011). UCHIME improves sensitivity and speed of chimera detection. *Bioinformatics* 27 2194–2200. 10.1093/bioinformatics/btr381 21700674 PMC3150044

[B22] Ellis S. Howe M. T. Goulding K. W. T. Mugglestone M. A. Dendooven L. (1998). Carbon and nitrogen dynamics in a grassland soil with varying pH: effect of pH on the denitrification potential and dynamics of the reduction enzymes. *Soil Biol. Biochem.* 30 359–367. 10.1016/S0038-0717(97)00122-3

[B23] Elshahed M. S. Youssef N. H. Spain A. M. Sheik C. Najar F. Z. Sukharnikov L. O. (2008). Novelty and uniqueness patterns of rare members of the soil biosphere. *Appl. Environ. Microbiol.* 74 5422–5428. 10.1128/AEM.00410-08 18606799 PMC2546616

[B24] Faílde L. D. Losada A. P. Bermúdez R. Santos Y. Quiroga M. I. (2013). Tenacibaculum maritimum infection: pathology and immunohistochemistry in experimentally challenged turbot (*Psetta maxima* L.). *Microb. Pathog.* 65 82–88. 10.1016/J.MICPATH.2013.09.003 24090541

[B25] Gaidos E. Rusch A. Ilardo M. (2011). Ribosomal tag pyrosequencing of DNA and RNA from benthic coral reef microbiota: community spatial structure, rare members and nitrogen-cycling guilds. *Environ. Microbiol.* 13 1138–1152. 10.1111/j.1462-2920.2010.02392.x 21176054

[B26] Galand P. E. Chapron L. Meistertzheim A.-L. Peru E. Lartaud F. (2018). The effect of captivity on the dynamics of active bacterial communities differs between two deep-sea coral species. *Front. Microbiol.* 9:2565. 10.3389/fmicb.2018.02565 30420844 PMC6215855

[B27] Gardner S. G. Nielsen D. A. Laczka O. Shimmon R. Beltran V. H. Ralph P. J. (2016). Dimethylsulfoniopropionate, superoxide dismutase and glutathione as stress response indicators in three corals under short-term hyposalinity stress. *Proc. R. Soc. B Biol. Sci.* 283:20152418. 10.1098/rspb.2015.2418 26865302 PMC4760162

[B28] Grottoli A. G. Martins P. D. Wilkins M. J. Johnston M. D. Warner M. E. Cai W. J. (2018). Coral physiology and microbiome dynamics under combined warming and ocean acidification. *PLoS One* 13:e0191156. 10.1371/journal.pone.0191156 29338021 PMC5770069

[B29] Hill R. Ralph P. J. (2006). Photosystem II heterogeneity of in hospite zooxanthellae in scleractinian corals exposed to bleaching conditions. *Photochem. Photobiol.* 82:1577. 10.1562/2006-04-13-RA-871 16961432

[B30] Hoegh-Guldberg O. (2007). Coral reefs under rapid climate change and ocean acidification. *Science* 318:1737. 10.1126/science.1137094 18079392

[B31] Hopkins F. Bell T. Yang M. Suggett D. Steinke M. (2016). Air exposure of coral is a significant source of dimethylsulfide (DMS) to the atmosphere. *Sci. Rep.* 6:36031. 10.1038/SREP36031 27796323 PMC5086842

[B32] Hoshino Y. T. Matsumoto N. (2007). DNA- versus RNA-based denaturing gradient gel electrophoresis profiles of a bacterial community during replenishment after soil fumigation. *Soil Biol. Biochem.* 39 434–444. 10.1016/J.SOILBIO.2006.08.013

[B33] Hughes T. Barnes M. L. Bellwood D. R. Cinner J. E. Cumming G. S. Jackson J. B. C. (2017). Coral reefs in the anthropocene. *Nature* 546:82. 28569801 10.1038/nature22901

[B34] Hunt D. E. Lin Y. Church M. J. Karl D. M. Tringe S. G. Izzo L. K. (2013). Relationship between abundance and specific activity of bacterioplankton in open ocean surface waters. *Appl. Environ. Microbiol.* 79 177–184. 10.1128/AEM.02155-12 23087033 PMC3536108

[B35] Huse S. M. Welch D. M. Morrison H. G. Sogin M. L. (2010). Ironing out the wrinkles in the rare biosphere through improved OTU clustering. *Environ. Microbiol.* 12:1889. 10.1111/J.1462-2920.2010.02193.X 20236171 PMC2909393

[B36] Jones S. E. Lennon J. T. (2010). Dormancy contributes to the maintenance of microbial diversity. *Proc. Natl. Acad. Sci. U.S.A.* 107 5881–5886. 10.1073/pnas.0912765107 20231463 PMC2851880

[B37] Jousset A. Bienhold C. Chatzinotas A. Gallien L. Gobet A. Kurm V. (2017). Where less may be more: how the rare biosphere pulls ecosystems strings. *ISME J.* 11 853–862. 10.1038/ismej.2016.174 28072420 PMC5364357

[B38] Kooperman N. Ben-Dov E. Kramarsky-Winter E. Barak Z. Kushmaro A. (2007). Coral mucus-associated bacterial communities from natural and aquarium environments. *FEMS Microbiol. Lett.* 276 106–113. 10.1111/j.1574-6968.2007.00921.x 17937669

[B39] Krediet C. J. Ritchie K. B. Paul V. J. Teplitski M. (2013). Coral-associated micro-organisms and their roles in promoting coral health and thwarting diseases. *Proc. R. Soc. B Biol. Sci.* 280:20122328. 10.1098/rspb.2012.2328 23363627 PMC3574386

[B40] LaJeunesse T. C. Parkinson J. E. Gabrielson P. W. Jeong H. J. Reimer J. D. Voolstra C. R. (2018). Systematic revision of symbiodiniaceae highlights the antiquity and diversity of coral endosymbionts. *Curr. Biol.* 28 2570–2580.e6. 10.1016/J.CUB.2018.07.008 30100341

[B41] Lane D. J. Pace B. Olsen G. J. Stahl D. A. Sogin M. L. Pace N. R. (1985). Rapid determination of 16S ribosomal RNA sequences for phylogenetic analyses. *Proc. Natl. Acad. Sci. U.S.A.* 82 6955–6959. 10.1073/pnas.82.20.6955 2413450 PMC391288

[B42] Lanzén A. Jørgensen S. L. Bengtsson M. M. Jonassen I. Øvreås L. Urich T. (2011). Exploring the composition and diversity of microbial communities at the jan mayen hydrothermal vent field using RNA and DNA. *FEMS Microbiol. Ecol.* 77 577–589. 10.1111/j.1574-6941.2011.01138.x 21627670

[B43] Lema K. A. Willis B. L. Bourneb D. G. (2012). Corals form characteristic associations with symbiotic nitrogen-fixing bacteria. *Appl. Environ. Microbiol.* 78 3136–3144. 10.1128/AEM.07800-11 22344646 PMC3346485

[B44] Lesser M. P. Mazel C. H. Gorbunov M. Y. Falkowski P. G. (2004). Discovery of symbiotic nitrogen-fixing cyanobacteria in corals. *Science* 305 997–1000. 10.1126/science.1099128 15310901

[B45] Li M.-Y. Yang Y.-H. Mi T.-Z. He H. Zhen Y. (2020). Differences between DNA- and RNA-based bacterial communities in marine sediments. *Huan Jing Ke Xue* 41 2485–2495. 10.13227/j.hjkx.201911071 32608868

[B46] Liew Y. J. Zoccola D. Li Y. Tambutté E. Venn A. A. Michell C. T. (2018). Epigenome-associated phenotypic acclimatization to ocean acidification in a reef-building coral. *Sci. Adv.* 4:eaar8028. 10.1126/sciadv.aar8028 29881778 PMC5990304

[B47] Lillis L. Doyle E. Clipson N. (2009). Comparison of DNA- and RNA-based bacterial community structures in soil exposed to 2,4-dichlorophenol. *J. Appl. Microbiol.* 107 1883–1893. 10.1111/j.1365-2672.2009.04369.x 20426769

[B48] Lynch M. D. J. Neufeld J. D. (2015). Ecology and exploration of the rare biosphere. *Nat. Rev. Microbiol.* 13 217–229. 10.1038/nrmicro3400 25730701

[B49] McCarthy A. Chiang E. Schmidt M. L. Denef V. J. (2015). RNA preservation agents and nucleic acid extraction method bias perceived bacterial community composition. *PLoS One* 10:e0121659. 10.1371/journal.pone.0121659 25798612 PMC4370824

[B50] McDonald D. Price M. N. Goodrich J. Nawrocki E. P. DeSantis T. Z. Probst A. (2012). An improved greengenes taxonomy with explicit ranks for ecological and evolutionary analyses of bacteria and archaea. *ISME J.* 6 610–618. 10.1038/ismej.2011.139 22134646 PMC3280142

[B51] Mengoni A. Tatti E. Decorosi F. Viti C. Bazzicalupo M. Giovannetti L. (2005). Comparison of 16S rRNA and 16S rDNA T-RFLP approaches to study bacterial communities in soil microcosms treated with chromate as perturbing agent. *Microb. Ecol.* 50 375–384. 10.1007/s00248-004-0222-4 16254761

[B52] Meron D. Atias E. Iasur Kruh L. Elifantz H. Minz D. Fine M. (2011). The impact of reduced pH on the microbial community of the coral *Acropora eurystoma*. *ISME J.* 5 51–60. 10.1038/ismej.2010.102 20668489 PMC3105665

[B53] Meron D. Rodolfo-Metalpa R. Cunning R. Baker A. C. Fine M. Banin E. (2012). Changes in coral microbial communities in response to a natural pH gradient. *ISME J.* 6 1775–1785. 10.1038/ismej.2012.19 22437157 PMC3498918

[B54] Moberg F. Folke C. (1999). Ecological goods and services of coral reef ecosystems. *Ecol. Econ.* 29 215–233. 10.1016/S0921-8009(99)00009-9

[B55] Morrow K. M. Bourne D. G. Humphrey C. Botté E. S. Laffy P. Zaneveld J. (2015). Natural volcanic CO2 seeps reveal future trajectories for host-microbial associations in corals and sponges. *ISME J.* 9 894–908. 10.1038/ismej.2014.188 25325380 PMC4817704

[B56] Neave M. J. Rachmawati R. Xun L. Michell C. T. Bourne D. G. Apprill A. (2017). Differential specificity between closely related corals and abundant *Endozoicomonas* endosymbionts across global scales. *ISME J.* 11 186–200. 10.1038/ismej.2016.95 27392086 PMC5335547

[B57] Nogales B. Moore E. R. Llobet-Brossa E. Rossello-Mora R. Amann R. Timmis K. N. (2001). Combined use of 16S ribosomal DNA and 16S rRNA to study the bacterial community of polychlorinated biphenyl-polluted soil. *Appl. Environ. Microbiol.* 67 1874–1884. 10.1128/AEM.67.4.1874-1884.2001 11282645 PMC92809

[B58] O’Brien P. A. Morrow K. M. Willis B. L. Bourne D. G. (2016). Implications of ocean acidification for marine microorganisms from the free-living to the host-associated. *Front. Mar. Sci.* 3:47. 10.3389/fmars.2016.00047

[B59] O’Brien P. A. Smith H. A. Fallon S. Fabricius K. Willis B. L. Morrow K. M. (2018). Elevated CO2 has little influence on the bacterial communities associated with the pH-tolerant coral, massive *Porites* spp. *Front. Microbiol.* 9:2621. 10.3389/fmicb.2018.02621 30443242 PMC6221987

[B60] Pedrós-Alió C. (2006). Marine microbial diversity: can it be determined? *Trends Microbiol.* 14 257–263. 10.1016/J.TIM.2006.04.007 16679014

[B61] Pedrós-Alió C. (2007). Dipping into the rare biosphere. *Science* 315 192–193. 10.1126/science.1136264 17218512

[B62] Pedrós-Alió C. (2012). The rare bacterial biosphere. *Ann. Rev. Mar. Sci.* 4 449–466. 10.1146/annurev-marine-120710-100948 22457983

[B63] Pietramellara G. Ascher J. Borgogni F. Ceccherini M. T. Guerri G. Nannipieri P. (2009). Extracellular DNA in soil and sediment: fate and ecological relevance. *Biol. Fert. Soils* 45 219–235. 10.1007/s00374-008-0345-8

[B64] Pogoreutz C. Rädecker N. Cárdenas A. Gärdes A. Voolstra C. R. Wild C. (2017a). Sugar enrichment provides evidence for a role of nitrogen fixation in coral bleaching. *Glob. Change Biol.* 23 3838–3848. 10.1111/gcb.13695 28429531

[B65] Pogoreutz C. Rädecker N. Cárdenas A. Gärdes A. Wild C. Voolstra C. R. (2017b). Nitrogen fixation aligns with nifH abundance and expression in two coral trophic functional groups. *Front. Microbiol.* 8:1187. 10.3389/fmicb.2017.01187 28702013 PMC5487474

[B66] Poulsen L. K. Ballard G. Stahl D. A. (1993). Use of rRNA fluorescence in situ hybridization for measuring the activity of single cells in young and established biofilms. *Appl. Environ. Microbiol.* 59 1354–1360. 7685999 10.1128/aem.59.5.1354-1360.1993 PMC182089

[B67] Pratte Z. A. Richardson L. L. Mills D. K. (2015). Microbiota shifts in the surface mucopolysaccharide layer of corals transferred from natural to aquaria settings. *J. Invertebr. Pathol.* 125 42–44. 10.1016/J.JIP.2014.12.009 25553581

[B68] Pruesse E. Quast C. Knittel K. Fuchs B. M. Ludwig W. Peplies J. (2007). SILVA: a comprehensive online resource for quality checked and aligned ribosomal RNA sequence data compatible with ARB. *Nucleic Acids Res.* 35 7188–7196. 10.1093/nar/gkm864 17947321 PMC2175337

[B69] Rädecker N. Pogoreutz C. Voolstra C. R. Wiedenmann J. Wild C. (2015). Nitrogen cycling in corals: the key to understanding holobiont functioning? *Trends Microbiol.* 23 490–497. 10.1016/J.TIM.2015.03.008 25868684

[B70] Raina J.-B. Tapiolas D. Willis B. L. Bourne D. G. (2009). Coral-associated bacteria and their role in the biogeochemical cycling of sulfur. *Appl. Environ. Microbiol.* 75 3492–3501. 10.1128/AEM.02567-08 19346350 PMC2687302

[B71] Ramos C. Mølbak L. Molin S. (2000). Bacterial activity in the rhizosphere analyzed at the single-cell level by monitoring ribosome contents and synthesis rates. *Appl. Environ. Microbiol.* 66 801–809. 10.1128/aem.66.2.801-809.2000 10653754 PMC91899

[B72] Reaka-Kudla M. L. (2001). “Known and unknown biodiversity, risk of extinction and conservation strategy in the sea,” in *Waters in Peril*, eds Bendell-Young L. Gallaugher P. (Boston, MA: Springer), 19–33. 10.1007/978-1-4615-1493-0_2

[B73] Rhein M. (2014). “Observations: ocean pages” in *Chapter 3, Observations: Oceans, in IPCC 2013, Climate Change 2013 - The Physical Science Basis* ed. Intergovernmental Panel on Climate Change (Cambridge: Cambridge University Press) 255–316. 10.1017/CBO9781107415324.010

[B74] Ritchie K. (2006). Regulation of microbial populations by coral surface mucus and mucus-associated bacteria. *Mar. Ecol. Prog. Ser.* 322 1–14. 10.3354/meps322001

[B75] Robbins S. J. Singleton C. M. Chan C. X. Messer L. F. Geers A. U. Ying H. (2019). A genomic view of the reef-building coral *Porites lutea* and its microbial symbionts. *Nat. Microbiol.* 4 2090–2100. 10.1038/s41564-019-0532-4 31548681

[B76] Roder C. Arif C. Daniels C. Weil E. Voolstra C. R. (2014). Bacterial profiling of white plague disease across corals and oceans indicates a conserved and distinct disease microbiome. *Mol. Ecol.* 23 965–974. 10.1111/mec.12638 24350609 PMC4285310

[B77] Rohwer F. Seguritan V. Azam F. Knowlton N. (2002). Diversity and distribution of coral-associated bacteria. *Mar. Ecol. Prog. Ser.* 243 1–10. 10.3354/meps243001

[B78] Romanowicz K. J. Freedman Z. B. Upchurch R. A. Argiroff W. A. Zak D. R. (2016). Active microorganisms in forest soils differ from the total community yet are shaped by the same environmental factors: the influence of pH and soil moisture. *FEMS Microbiol. Ecol.* 92:fiw149. 10.1093/femsec/fiw149 27387909

[B79] Rosenberg E. (2013). *The Prokaryotes: Alphaproteobacteria and Betaproteobacteria.* Berlin: Springer, 1–1012. 10.1007/978-3-642-30197-1

[B80] Rosenberg E. Koren O. Reshef L. Efrony R. Zilber-Rosenberg I. (2007). The role of microorganisms in coral health, disease and evolution. *Nat. Rev. Microbiol.* 5 355–362. 10.1038/nrmicro1635 17384666

[B81] Röthig T. Roik A. Yum L. K. Voolstra C. R. (2017). Distinct bacterial microbiomes associate with the Deep-Sea Coral *Eguchipsammia fistula* from the red sea and from aquaria settings. *Front. Mar. Sci.* 4:259. 10.3389/fmars.2017.00259

[B82] Salgar-Chaparro S. J. Machuca L. L. (2019). Complementary DNA/RNA-based profiling: characterization of corrosive microbial communities and their functional profiles in an oil production facility. *Front. Microbiol.* 10:2587. 10.3389/fmicb.2019.02587 31787960 PMC6853844

[B83] Schloss P. D. Westcott S. L. Ryabin T. Hall J. R. Hartmann M. Hollister E. B. (2009). Introducing mothur: open-source, platform-independent, community–supported software for describing and comparing microbial communities. *Appl. Environ. Microbiol.* 75 7537–7541. 10.1128/AEM.01541-09 19801464 PMC2786419

[B84] Schöttner S. Hoffmann F. Wild C. Rapp H. T. Boetius A. Ramette A. (2009). Inter- and intra-habitat bacterial diversity associated with cold-water corals. *ISME J.* 3 756–759. 10.1038/ismej.2009.15 19279671

[B85] Segata N. Izard J. Waldron L. Gevers D. Miropolsky L. Garrett W. S. (2011). Metagenomic biomarker discovery and explanation. *Genome Biol.* 12:R60. 10.1186/gb-2011-12-6-r60 21702898 PMC3218848

[B86] Shade A. Handelsman J. (2012). Beyond the venn diagram: the hunt for a core microbiome. *Environ. Microbiol.* 14 4–12. 10.1111/j.1462-2920.2011.02585.x 22004523

[B87] Sogin M. L. Morrison H. G. Huber J. A. Mark Welch D. Huse S. M. Neal P. R. (2006). Microbial diversity in the deep sea and the underexplored &quot;rare biosphere&quot. *Proc. Natl. Acad. Sci. U.S.A.* 103 12115–12120. 10.1073/pnas.0605127103 16880384 PMC1524930

[B88] Steven B. Hesse C. Soghigian J. Gallegos-Graves L. V. Dunbar J. (2017). Simulated ribosomal RNA:DNA ratios show potential to misclassify active populations as dormant. *Appl. Environ. Microbiol.* 83 e00696–17. 10.1128/AEM.00696-17 28363969 PMC5440720

[B89] Sussman A. S. Douthit H. A. (1973). *Dormancy in Microbial Spores.* Available online at: www.annualreviews.org (accessed February 27, 2021)

[B90] Tambutté E. Venn A. A. Holcomb M. Segonds N. Techer N. Zoccola D. (2015). Morphological plasticity of the coral skeleton under CO2-driven seawater acidification. *Nat. Commun.* 6:7368. 10.1038/ncomms8368 26067341 PMC4490415

[B91] Thörn M. Sörensson F. (1996). Variation of nitrous oxide formation in the denitrification basin in a wastewater treatment plant with nitrogen removal. *Water Res.* 30 1543–1547. 10.1016/0043-1354(95)00327-4

[B92] Thurber R. V. Willner-Hall D. Rodriguez-Mueller B. Desnues C. Edwards R. A. Angly F. (2009). Metagenomic analysis of stressed coral holobionts. *Environ. Microbiol.* 11 2148–2163. 10.1111/j.1462-2920.2009.01935.x 19397678

[B93] Tilstra A. El-Khaled Y. C. Roth F. Rädecker N. Pogoreutz C. Voolstra C. R. (2019). Denitrification aligns with N2 fixation in red sea corals. *Sci. Rep.* 9:19460. 10.1038/s41598-019-55408-z 31857601 PMC6923481

[B94] Venn A. A. Tambutté E. Holcomb M. Laurent J. Allemand D. Tambutté S. (2013). Impact of seawater acidification on pH at the tissue–skeleton interface and calcification in reef corals. *Proc. Natl. Acad. Sci. U.S.A.* 110 1634–1639. 10.1073/PNAS.1216153110 23277567 PMC3562847

[B95] Voolstra C. R. Ziegler M. (2020). Adapting with microbial help: microbiome flexibility facilitates rapid responses to environmental change. *Bioessays* 42:2000004. 10.1002/bies.202000004 32548850

[B96] Webster N. S. Negri A. P. Botté E. S. Laffy P. W. Flores F. Noonan S. (2016). Host-associated coral reef microbes respond to the cumulative pressures of ocean warming and ocean acidification. *Sci. Rep.* 6:19324. 10.1038/srep19324 26758800 PMC4725835

[B97] Webster N. S. Negri A. P. Flores F. Humphrey C. Soo R. Botté E. S. (2013). Near-future ocean acidification causes differences in microbial associations within diverse coral reef taxa. *Environ. Microbiol. Rep.* 5 243–251. 10.1111/1758-2229.12006 23584968

[B98] Weigold P. Ruecker A. Loesekann-Behrens T. Kappler A. Behrens S. (2016). Ribosomal tag pyrosequencing of DNA and RNA reveals “rare” taxa with high protein synthesis potential in the sediment of a hypersaline lake in Western Australia. *Geomicrobiol. J.* 33 426–440. 10.1080/01490451.2015.1049304

[B99] Weisburg W. G. Barns S. M. Pelletier D. A. Lane D. J. (1991). 16S ribosomal DNA amplification for phylogenetic study. *J. Bacteriol.* 173 697–703. 10.1128/jb.173.2.697-703.1991 1987160 PMC207061

[B100] Wright R. M. Kenkel C. D. Dunn C. E. Shilling E. N. Bay L. K. Matz M. V. (2017). Intraspecific differences in molecular stress responses and coral pathobiome contribute to mortality under bacterial challenge in *Acropora millepora*. *Sci. Rep.* 7:2609. 10.1038/s41598-017-02685-1 28572677 PMC5454005

[B101] Yang S. Sun W. Zhang F. Li Z. (2013). Phylogenetically diverse denitrifying and ammonia-oxidizing bacteria in corals *Alcyonium gracillimum* and *Tubastraea coccinea*. *Mar. Biotechnol.* 15 540–551. 10.1007/s10126-013-9503-6 23564007

[B102] Zhou G. Yuan T. Cai L. Zhang W. Tian R. Tong H. (2016). Changes in microbial communities, photosynthesis and calcification of the coral *Acropora gemmifera* in response to ocean acidification. *Sci. Rep.* 6:35971. 10.1038/srep35971 27786309 PMC5082368

