# Supplementary material for: Effects of Ocean Acidification on Resident and Active Microbial Communities of Stylophora pistillata
Source: Front Microbiol. 2021 Nov 25;12:707674. doi: 10.3389/fmicb.2021.707674 (PMC8656159; doi:10.3389/fmicb.2021.707674)
Supplement: Supplementary file 1 [file Table_1.DOCX]

**Supplementary tables and figures**

Supplementary table 1. Number of sequences before and after quality filtering for samples from *Stylophora pistillata* kept in aquaria for two years at different pH treatments.

| Samples |  | pH | Before quality filtering | After quality filtering |
| --- | --- | --- | --- | --- |
| C01 | RNA | 7.2 | 127,555 | 41,955 |
| C02 | RNA | 7.2 | 146,932 | 50,788 |
| C03 | RNA | 7.2 | 145,654 | 44,298 |
| C04 | RNA | 7.4 | 213,481 | 90,463 |
| C05 | RNA | 7.4 | 137,859 | 64,326 |
| C06 | RNA | 7.4 | 257,126 | 114,558 |
| C07 | RNA | 7.8 | 85,043 | 24,287 |
| C08 | RNA | 7.8 | 100,685 | 40,672 |
| C09 | RNA | 7.8 | 34,450 | 16,403 |
| C10 | RNA | 8.0 | 42,315 | 10,669 |
| C11 | RNA | 8.0 | 126,502 | 38,964 |
| C12 | RNA | 8.0 | 65,379 | 19,407 |
| G01 | DNA | 7.2 | 212,468 | 67,405 |
| G02 | DNA | 7.2 | 146,925 | 50,691 |
| G03 | DNA | 7.2 | 166,924 | 59,933 |
| G04 | DNA | 7.4 | 428,649 | 165,630 |
| G05 | DNA | 7.4 | 116,491 | 38,031 |
| G06 | DNA | 7.4 | 275,208 | 103,276 |
| G07 | DNA | 7.8 | 202,930 | 76,129 |
| G08 | DNA | 7.8 | 215,813 | 61,335 |
| G09 | DNA | 7.8 | 45,251 | 13,735 |
| G10 | DNA | 8.0 | 217,604 | 76,125 |
| G11 | DNA | 8.0 | 380,343 | 127,074 |
| G12 | DNA | 8.0 | 266,332 | 78,495 |

Supplementary table 2. Presence and absence of most important bacterial OTUs in DNA and RNA samples in samples from *Stylophora pistillata* keept in acquaria for two years at different pH treatments (pH 7.2, 7.4, 7.8 and 8).

| Most abundant DNA OTUs | | 7.2 DNA | 7.2 RNA | 7.4 DNA | 7.4 RNA | 7.8 DNA | 7.8 RNA | 8.0 DNA | 8.0 RNA |
| --- | --- | --- | --- | --- | --- | --- | --- | --- | --- |
| Otu0002 | Thalassotalea | Present | Present | Present | Present | Present | Present | Present | Present |
| Otu0004 | Alteromonas | Present | Present | Present | Present | Present | Present | Present | Present |
| Otu0005 | Flavobacteriaceae | Present | Present | Present | Absent | Present | Present | Present | Present |
| Otu0007 | Prosthecochloris | Present | Present | Present | Absent | Present | Present | Present | Absent |
| Otu0008 | Alteromonas | Present | Present | Present | Absent | Present | Present | Present | Present |
| Otu0012 | Thalassolituus | Present | Present | Present | Absent | Present | Present | Present | Present |
| Otu0011 | Kordia | Present | Present | Present | Present | Present | Present | Present | Present |
| Otu0013 | Shimia | Present | Present | Present | Absent | Present | Present | Present | Present |
| Otu0014 | Thalassobius | Present | Present | Present | Present | Present | Present | Present | Present |
| Otu0001 | Fulvivirga | Present | Present | Present | Present | Present | Present | Present | Present |
| Most abundant RNA OTUs | | **7.2 DNA** | **7.2 RNA** | **7.4 DNA** | **7.4 RNA** | **7.8 DNA** | **7.8 RNA** | **8.0 DNA** | **8.0 RNA** |
| Otu0001 | Flammeovirgaceae | Present | Present | Present | Present | Present | Present | Present | Present |
| Otu0003 | Candidatus amoebophilus | Present | Present | Present | Present | Present | Present | Present | Present |
| Otu0006 | Cellvibrionaceae | Present | Present | Present | Present | Present | Absent | Present | Present |
| Otu0009 | Castellaniella | Present | Present | Present | Present | Present | Present | Present | Present |
| Otu0015 | Candidatus amoebophilus | Absent | Absent | Absent | Present | Absent | Present | Absent | Present |
| Otu0016 | Cytophagales | Absent | Absent | Absent | Present | Absent | Present | Absent | Present |
| Otu0017 | Francisellaceae | Present | Present | Present | Present | Present | Present | Present | Present |
| Otu0011 | Kordia | Present | Present | Present | Present | Present | Present | Present | Present |
| Otu0013 | Shimia | Present | Present | Present | Absent | Present | Present | Present | Present |
| Otu0014 | Thalassobius | Present | Present | Present | Present | Present | Present | Present | Present |
| Core Microbiome | | **RNA 7.2** | **RNA 7.4** | **RNA 7.8** | **RNA 8.0** |  |  |  |  |
| Otu0001 | Fulvivirga | Present | Present | Present | Present |  |  |  |  |
| Otu0002 | Thalassotalea | Present | Present | Present | Present |  |  |  |  |
| Otu0004 | Alteromonas | Present | Present | Present | Present |  |  |  |  |
| Otu0005 | Flavobacteriaceae | Present | Absent | Present | Present |  |  |  |  |
| Otu0008 | Alteromonas | Present | Absent | Present | Present |  |  |  |  |
| Otu0010 | Tenacibaculum | Present | Present | Present | Present |  |  |  |  |
| Otu0012 | Thalassolituus | Present | Absent | Present | Present |  |  |  |  |
| Otu0011 | Kordia | Present | Present | Present | Present |  |  |  |  |
| Otu0013 | Shimia | Present | Absent | Present | Present |  |  |  |  |
| Otu0014 | Thalassobius | Present | Present | Present | Present |  |  |  |  |
| Otu0018 | Salinimonas | Present | Absent | Present | Present |  |  |  |  |
| Otu0020 | Tenacibaculum | Present | Absent | Present | Present |  |  |  |  |
| Otu0019 | Unclassified bacteria | Present | Present | Present | Present |  |  |  |  |
| Otu0023 | Oleiphilus | Present | Present | Present | Present |  |  |  |  |
| Otu0024 | Pelomonas | Present | Present | Present | Present |  |  |  |  |
| Otu0025 | Cutibacterium | Present | Present | Present | Present |  |  |  |  |
| Otu0027 | Pseudoalteromonas | Present | Absent | Absent | Absent |  |  |  |  |
| Otu0028 | Flavobacteriaceae | Absent | Absent | Absent | Present |  |  |  |  |
| Otu0031 | Endozoicomonas | Absent | Present | Absent | Absent |  |  |  |  |
| Otu0032 | Vibrio | Present | Present | Present | Present |  |  |  |  |
| Otu0039 | Microscilla | Present | Absent | Present | Present |  |  |  |  |
| Otu0043 | Ruegeria | Present | Present | Present | Present |  |  |  |  |
| Otu0095 | Rhodobacteraceae | Present | Present | Present | Present |  |  |  |  |

Supplementary table 3. Similarity values found for samples from *Stylophora pistillata* keept in acquaria for two years at different pH treatments (pH 7.2, 7.4, 7.8 and 8.0) a) using RNA subset; b) using DNA subset. Values marked in red and black indicate average similarity and dissimilarity values, respectively. All values shown are in percentage.

| **a)** RNA | | | | | **b)** DNA | | | | |  |  |
| --- | --- | --- | --- | --- | --- | --- | --- | --- | --- | --- | --- |
| pH | 7.2 | 7.4 | 7.8 | 8 | pH | 7.2 | 7.4 | 7.8 | 8 | DNA | RNA |
| 7.2 | 41.56 | 65.10 | 77.41 | 86.78 | 7.2 | 47.37 | 65.32 | 67.00 | 74.91 | 42.27 | 87.14 |
| 7.4 |  | 50.03 | 65.08 | 88.34 | 7.4 |  | 42.54 | 67.28 | 62.38 |  | 39.56 |
| 7.8 |  |  | 31.59 | 82.82 | 7.8 |  |  | 32.44 | 67.07 |  |  |
| 8 |  |  |  | 35.07 | 8 |  |  |  | 46.73 |  |  |

Supplementary table 4. Most important OTUs contributing to dissimilarity between RNA and DNA libraries in samples from *Stylophora pistillata* keept in acquaria for two years at different pH treatments (pH 7.2, 7.4, 7.8 and 8.0).

|  |  | RNA | DNA |  |  |  |  |
| --- | --- | --- | --- | --- | --- | --- | --- |
| Species |  | Av.Abund | Av.Abund | Av.Diss | Diss/SD | Contrib% | Cum.% |
| Otu0004 | Alteromonas | 1.32 | 7.18 | 0.79 | 2.21 | 0.90 | 0.90 |
| Otu0002 | Thalassotalea | 2.03 | 7.53 | 0.76 | 1.95 | 0.87 | 1.77 |
| Otu0010 | Tenacibaculum | 0.79 | 5.86 | 0.69 | 2.10 | 0.79 | 2.57 |
| Otu0008 | Alteromonas | 0.77 | 5.87 | 0.68 | 2.31 | 0.78 | 3.35 |
| Otu0005 | Unclassified Flavobacteriaceae | 0.95 | 6.08 | 0.68 | 1.83 | 0.78 | 4.13 |
| Otu0012 | Thalassolituus | 0.62 | 5.42 | 0.67 | 1.88 | 0.77 | 4.89 |
| Otu0003 | Candidatus amoebophilus | 7.03 | 2.31 | 0.64 | 1.75 | 0.73 | 5.63 |
| Otu0001 | Fulvivirga | 7.80 | 4.11 | 0.61 | 1.54 | 0.70 | 6.33 |
| Otu0018 | Salinimonas | 1.00 | 4.60 | 0.51 | 1.74 | 0.58 | 6.91 |
| Otu0020 | Tenacibaculum | 0.45 | 4.08 | 0.49 | 1.99 | 0.56 | 7.47 |
| Otu0023 | Oleiphilus | 0.87 | 4.28 | 0.47 | 1.87 | 0.54 | 8.01 |
| Otu0013 | Shimia | 2.06 | 5.11 | 0.47 | 1.35 | 0.54 | 8.55 |
| Otu0011 | Kordia | 2.21 | 4.71 | 0.43 | 1.33 | 0.49 | 9.05 |
| Otu0015 | Candidatus amoebophilus | 3.19 | 0.00 | 0.43 | 0.87 | 0.49 | 9.54 |
| Otu0014 | Thalassobius | 2.22 | 5.16 | 0.42 | 1.48 | 0.49 | 10.02 |
| Otu0030 | Aestuariibacter | 0.06 | 3.33 | 0.42 | 1.78 | 0.48 | 10.50 |
| Otu0028 | Unclassified Flavobacteriaceae | 0.28 | 3.28 | 0.40 | 1.21 | 0.46 | 10.96 |
| Otu0016 | Unclassified Cytophagales | 2.90 | 0.00 | 0.39 | 0.84 | 0.45 | 11.41 |
| Otu0021 | Psychrosphaera | 0.12 | 3.00 | 0.38 | 1.15 | 0.44 | 11.85 |
| Otu0027 | Pseudoalteromonas | 0.06 | 2.65 | 0.37 | 1.23 | 0.43 | 12.28 |
| Otu0031 | Endozoicomonas | 0.06 | 2.88 | 0.36 | 1.48 | 0.41 | 12.69 |


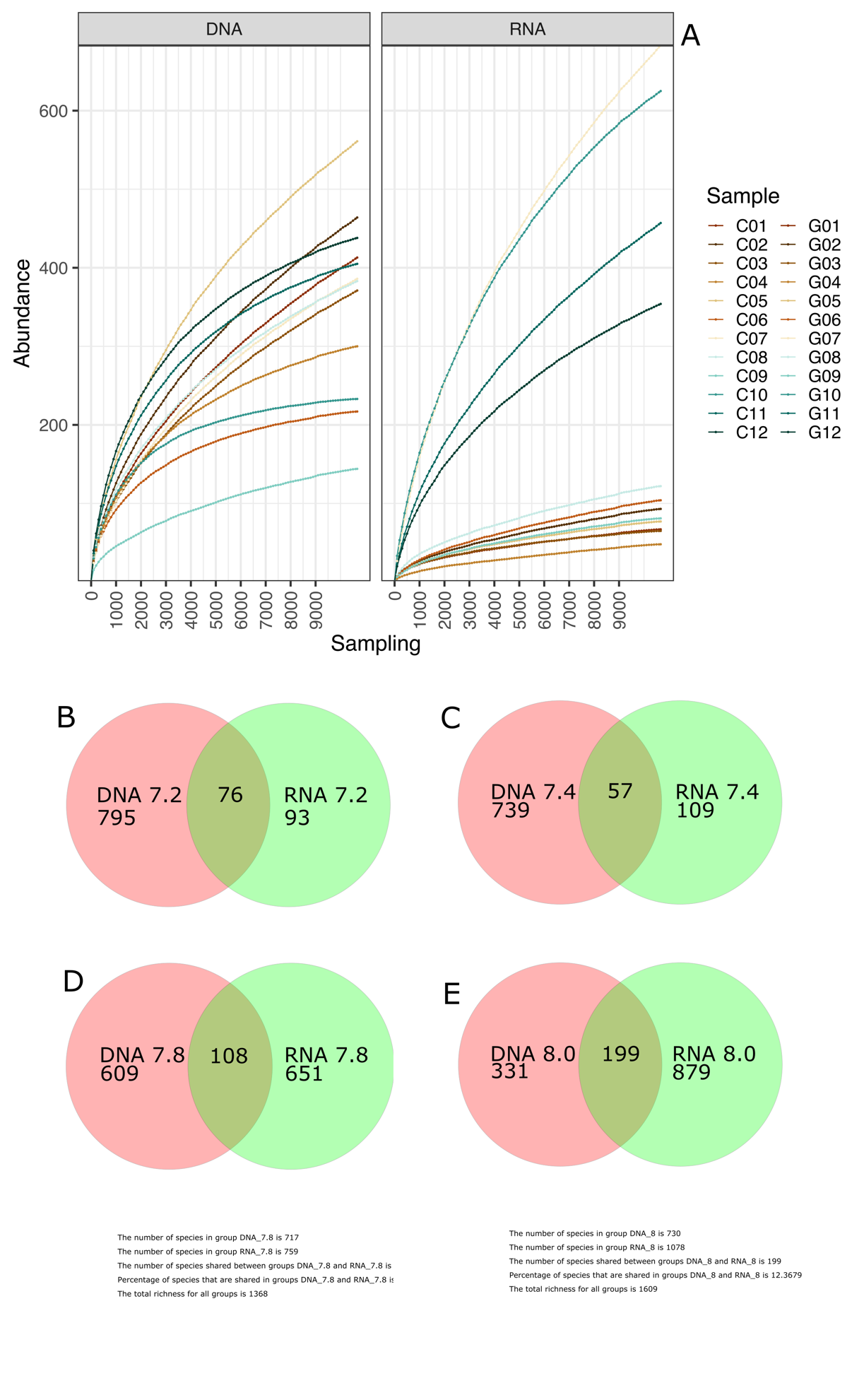


Supplementary figure 1. (A) Rarefaction curves for DNA and RNA samples from *Stylophora pistillata* kept in aquaria for two years at different pH treatments (pH 7.2, 7.4, 7.8 and 8.0), after subsampling to 10,669 reads. Three replicates from each pH treatment were sampled, C01-C03/=pH 7.2; C04-C06=pH 7.4; C07-C09=pH 7.8; C10-C12=pH 8.0 for RNA and G01-G03=pH 7.2; G04-G06=pH 7.4; G07-G09=pH 7.8; G10-G12=pH 8 for DNA; (B) Venn diagram showing *Stylophora pistillata* microbial shared and exclusive OTUs between DNA and RNA samples for pH 7.2; (C) pH 7.4; (D) pH 7.8 and (E) pH 8.0.


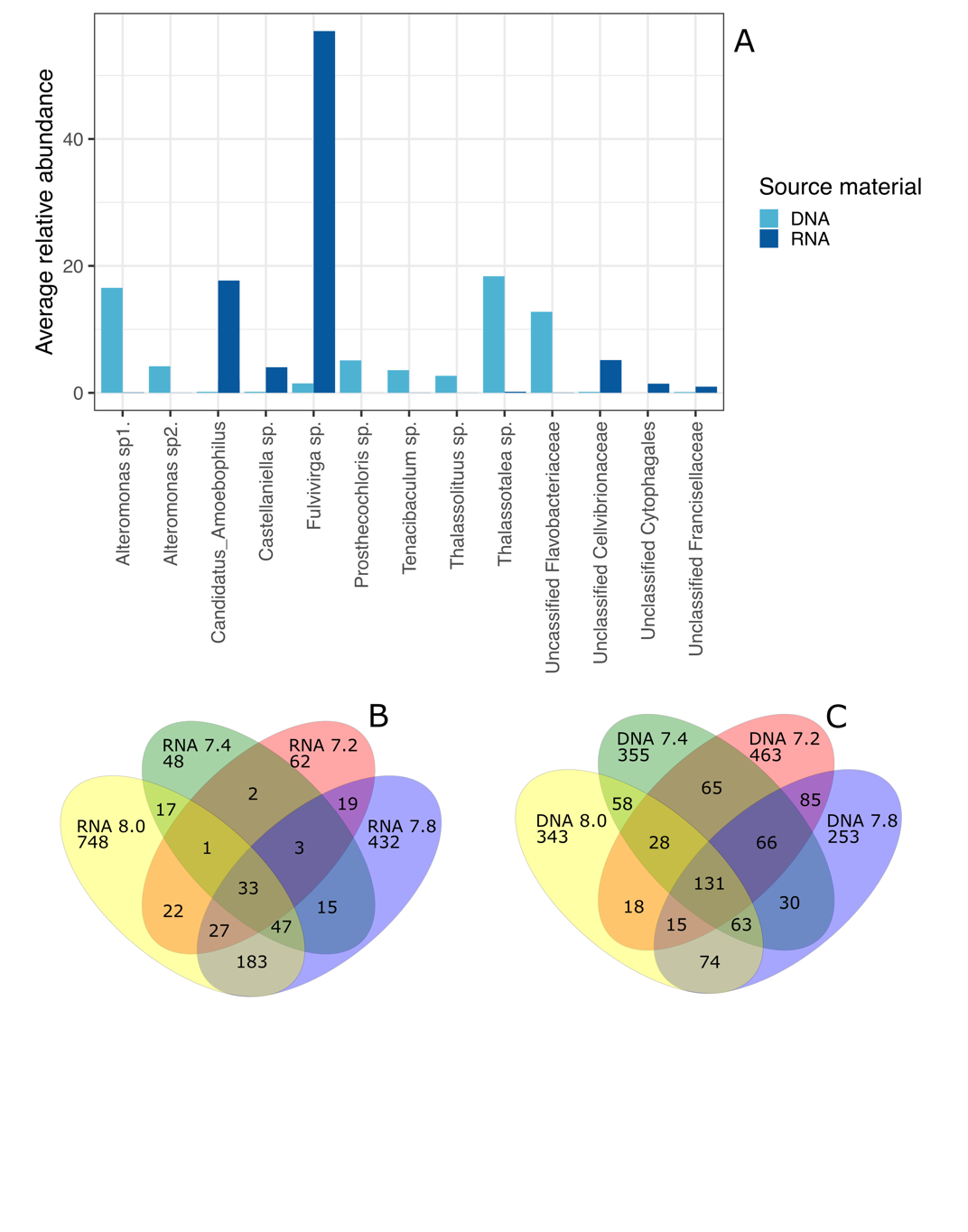


Supplementary figure 2. (A) Difference in relative abundance of most important microbial taxa between RNA and DNA libraries for *Stylophora pistillata* kept in aquaria for two years at different pH treatments (pH 7.2, 7.4, 7.8 and 8); (B) Venn diagram showing *Stylophora pistillata* microbial shared OTUs between different pH treatments (pH 7.2, 7.4, 7.8 and 8.0) for DNA and (C) RNA sample
